# Perspectives of key stakeholders on educational experiences of children with autism spectrum disorders at the Kenyan Coast

**DOI:** 10.4102/ajod.v11i0.847

**Published:** 2022-02-23

**Authors:** Amina Abubakar, Joseph K. Gona, Patricia Kipkemoi, Kenneth Rimba, Dennis Amukambwa, Charles R.J.C. Newton

**Affiliations:** 1 Neurosciences Research Group, Centre for Geographic Medicine Research-Coast, KEMRI-Welcome Trust Research Programme, Kilifi, Kenya; 2 Institute for Human Development, Aga Khan University, Nairobi, Kenya; 3 Department of Psychiatry, University of Oxford, Oxford, United Kingdom

**Keywords:** autism spectrum disorders, education, assessment, teacher training, special needs

## Abstract

**Background:**

Little is known about the educational experiences of children diagnosed with autism spectrum disorders (ASDs) in the Kenyan Coastal context.

**Objectives:**

We examined the diagnostic and placement procedures used in education on the Kenyan coastal region. In addition, we investigated the education-related challenges faced by children with ASD.

**Methods:**

We conducted focus group discussions and in-depth interviews with 21 participants, including teachers, clinicians and educational administrators. Data were analysed using an inductive thematic framework on qualitative data analysis software, NVIVO 10.

**Results:**

The findings from this study indicate that there were no systematic approaches to diagnosing children as having ASD. Teachers reported experiencing many challenges, including a lack of specialised training, inadequate resources and difficulty in managing children with different functional abilities in one class.

**Conclusion:**

There is an urgent need for contextually relevant evidence-based identification, placement and management services to be put in place to meet the educational needs of children with ASD.

## Introduction

The prevalence and impact of autism spectrum disorders (ASDs) in sub-Saharan Africa (SSA) remain unknown (Abubakar et al. 2016a). Recent studies reviewing or estimating the global burden of ASD point to the need for more data from low- and middle-income countries (Damiano & Forssberg 2019; Olusanya et al. 2018). In recent years, there has been increased efforts to understand the prevalence of ASD in SSA (Kakooza-Mwesige et al. 2014), validate screening and diagnostic tools for ASD (Harrison et al. 2014; Kakooza-Mwesige et al. 2014), understand the risk factors and markers for ASD, understand the psychosocial factors influencing the lives of children with ASD (Gona et al. 2015) and preliminary efforts at developing interventions (Franz et al. 2018). However, there has been little published work on the educational experiences and learning context for children diagnosed with ASD (Abubakar, Ssewanyana & Newton 2016b).

Two unpublished works from Kenya (Cohen 2012; Riccio 2011) reported some of the challenges faced by children with ASD and their families in Nairobi and Western Regions of Kenya. These studies used informal interview techniques with a range of stakeholders and observed that children with ASD experienced a host of challenges within the educational setting. These challenges include misunderstanding about the potential of autistic children and the lack of individual attention in the classroom, all of which affects their learning and development potential. However, these were small-scale studies that did not use a systematic methodology, and the extent to which these results can be generalised to other regions in the country remains to be established.

Schools form an important management and educational care centre for children with ASD in many parts of the world (Marsh et al. 2017). Following a diagnosis of ASD, most children with substantial and very substantial levels of support needs will need individualised education plans for them to acquire the necessary skills and knowledge to help them and their families cope with the day-to-day needs of their condition (Rabba et al. 2019; Vasilevska Petrovska et al. 2021). Most ASD screening and diagnostic tools are not widely used in the SSA context. They are costly, time-consuming, and require some expertise and training to administer and interpret the data (Divan et al. 2021). An additional complexity to diagnosing ASD in SSA is a medical model limiting diagnosis to few medical experts, such as psychiatrists and psychologists (Abubakar et al. 2016a). In countries like India, teachers with master’s level education are trained to use screening and diagnostic tools to make reliable diagnoses (Bhavnani et al. 2021). In order to optimise the school experience and outcomes of children with ASD, there is a need for evidence-based identification and programmes. As a first step towards this aim, we carried out a study to examine the current situation in Kenyan schools as they relate to ASD.

Within the Kenyan Coastal educational system, the Educational Assessment and Resource Centres (EARCs) are mandated by the Ministry of Education to diagnose and provide placement guidelines for children with special needs. At present, there are no validated tools for the screening and diagnosis of ASD in East Africa for use by either healthcare workers or teachers. This further contributes to the inequity in diagnosis and management of ASD in SSA. Thus far, there have been no systematic studies examining how diagnosis is made, the process of placement, or the challenges faced by children and caretakers of children with ASD in Kenya. Given the lack of research evidence, we set out to examine the educational experiences of children with ASD at the Kenyan Coast. Specifically, we set out to answer the following research questions: ‘What are the challenges faced by teachers working with children with ASD in the educational context, according to key stakeholders?’

## Methods

### Study site

The study was based at the Centre for Geographic Medicine Research in Kilifi County, Kenya, which covers an area of 12 610 km^2^ with an estimated population of 1 109 735 people. The languages spoken by a majority of the people include Kigiriama and Kiswahili. At the time of data collection, the county had a total of nine schools for children with intellectual and or socio-behavioural problems. Eight of these schools had special units for children with intellectual disabilities, including those with autism. Of the nine schools, only one has a class exclusively educating children with autism.

### Sample size and sampling procedures

We collected data using focus group discussions (FGDs) and in-depth interviews. The use of both methods was largely for convenience as some participants were not able to attend FGD sessions, and thus, in-depth interview at their convenience was the best way forward. We held two FGDs, one was with clinicians (*n* = 5) and the other one with teachers (*n* = 7). We also held nine in-depth interviews with key informants who have contact with parents with autistic children in various capacities. The children had a presumptive diagnosis of ASD from the EARC, where an EARC officer administered a questionnaire from the Kenya Institute of Special Education, which included questions on ASD symptomatology. They included non-governmental organisation (NGO) staff involved in disability support and advocacy work, educational administrators and EARC officers, who are part of the assessment team involved in the screening and placement of children in the special education system. Therefore, a total of 21 professionals (females, *n* = 7, 33%) took part in this study.

### Data collection

Following informed consent for participation, participants were interviewed or took part in the FGDs at venues that were most convenient for them during the data collection phase in 2013. All the interviews and FGDs were facilitated by the second author (J.K.G.) in Kiswahili, Kigiriama or English languages. All interviews and FGDs were audiotaped. A set of questions guided the sessions to ensure consistency. Probes and clarifications were sought as deemed necessary.

### Interview tool

A checklist of questions was developed by the research team through discussion and consensus. Refinement occurred based upon the initial interviews and discussion of the initial transcripts. Table 1 presents the interview schedule used. The questions core to the interviews are presented; however, probes were introduced to clarify and enhance the quality of the interviews.

**TABLE 1:** Sample questions in the interview schedule.†

Number	Question
1.	What do you think are the challenges faced by teachers teaching children with ASD?
2.	How do you identify these children in your assessment here? Do you have tools?
3.	What training have you received to be able to teach such children?
4.	What type of tools do you use in identifying them during your assessment?
5.	What challenges do you go through when helping these children in the classroom?

ASD, autism spectrum disorder.

† , Question wording was changed based on the target group to make them appropriate.

### Data management and analysis

The final transcripts used for analysis were based on the audio-taped materials. Data were analysed using NVIVO 10 (QSR International; New York) by the framework analysis (Silverman 2010; Strauss & Corbin 1998). The transcripts of the interviews were reviewed and read (familiarisation), during which a coding scheme was developed. The first three authors (A.A., J.K.G. and P.K.) worked together to develop the coding scheme and cross-validate the data. Themes were derived inductively, conflicting ideas were discussed, and consensus was reached.

### Ethical considerations

This study was approved on 15 August 2012 by the Kenya Medical Research Institute National Ethics and Review Committee, reference number: KEMRI/RES/7/3/1. Written informed consents were obtained from the participants. It was emphasised that participation in the study was voluntary, and there were no subsequent consequences for refusal or withdrawal.

## Results

Our data identified five key educational challenges faced by children with ASD at the Coast. They include (1) inadequate diagnostic, identification and placement procedures; (2) lack of specialised trained personnel; (3) heavy workload for the teachers; (4) lack of social support for the teachers; and (5) inadequate educational materials at the centres.

### Inadequate diagnostic, identification and placement procedures

Participants mentioned various diagnostic methods for ASD. They included what was referred to as ‘clinical observation’, a review of developmental history, performance-based assessment, questionnaires, and a combination of parental reports and observations:

‘There are signs and symptoms that are in the books that you are supposed to pick like any other disease that we learn. So, there are specific things that you need to tease out. … mainly it is the clinical diagnosis.’ (FGD, Clinical Officer, Participant 3, Male)

The teachers reported that by observing the children, they detect symptoms of ASD, and use this to identify a child as having ASD:

‘I can identify them through the outstanding behaviours they have, which are different from other people. For example, the habit of consistently doing a certain activity ….’ (Interview 13, special education teacher, Male)

Some of the educationalists mentioned that they would use the combination of both observation and parent-report questionnaires to identify children exhibiting autistic traits. During the discussions, we requested a copy of some of the measures. None of the measures used were directly designed to detect autistic features, and none had been validated or normed for use in this context.

Our discussions with the various stakeholders highlighted that there were no systematic approaches or guidelines on how to diagnose children having ASD. Related to the lack of diagnostic tools was the obvious lack of guidelines on placement. The lack of strategies for early diagnosis and placement is a major drawback for families of children with ASD and other neurodevelopmental disorders. It emerged from the discussions that there was a general perception, especially amongst the clinicians, that in rural areas most children were diagnosed as having ‘ASD’ whilst attending school. The clinicians noted that waiting for the child with developmental problems to be identified in the education system is problematic as it means that the children miss out the possibility of early diagnosis and intervention:

‘I think if we wait for teachers to make a diagnosis for us, it will be too late. I think the community should be given the information much earlier because, for those of us who live in town, we take our children to school (preschool) when they are 2 years old but back in the village the child will go to school when they are 7 years old. So, you can imagine a 7 years old child of, these symptoms would have shown much earlier ….’ (FGD, Clinical Officer, Participant 1, Male)

The statement above led to further discussion on the potential role of Mother–Child Health (MCH) clinics. Mother–Child Health clinicsare postnatal clinics, where parents are encouraged to take their children to receive vaccinations and monitor their growth and development until the age of 5 years. In the discussion by clinicians, it was noted that MCH has the potential to detect children with ASD and other neurodevelopmental disorders. However, as the workload is usually heavy, the child’s development is not assessed – hence, the opportunity for early diagnosis is lost:

‘Let me add something when it comes to MCH, the clinic has more than 100 patients queuing, and for you to diagnose a disease, you need to have enough time to examine that child. Basically, what people are doing at the MCH is forwarding and clearing, the queue is so huge, you alone are seeing 100 patients, so you check on the symptoms that have brought that child to the hospital that day. You are not examining the child holistically and trying to unravel what has not been told, so if we can increase the personnel, for people to have time and understand that we need to look at the child holistically, then we will be able to capture some of these symptoms. As for now, for one to be diagnosed with ASD, it will have taken the time and they would have moved from hospital to hospital ….’ (FGD, Clinical Officer, Participant 1, Male)

### Lack of specialised trained personnel

Many of those interviewed (13/21) participants expressed the view that most of the teachers in special education schools and units lacked proper training to meet the educational needs of children with ASD:

‘I have not gotten any special training, but it is out of interest that I cooperate with Mr. XXXX. I am interested in helping them, but I have not attended any course related to them (children with ASD) ….’ (Interview 3, Special Education Teacher, Female)

The lack of proper training was considered to be a problem not just for the teachers but also for others who are working in the educational sector, such as those in charge of assessing and giving a diagnosis for the children:

‘The challenges that teachers are facing and even (I) personally am facing is to know exactly what is to be done with these children because as I have said, we have just undergone a kind of general course. If we were to get training particularly on autism, exactly what is to be done with these children, then we would have good progress ….’ (Interview 12, Educational Assessor, Male)

### Inadequate educational materials at the centres

Other than the lack of trained personnel, the other challenge mentioned included the lack of teaching aids:

‘For those children (with autism), they need toys and play activities; they need puzzles, the wooden ones. Those are needed in their school but most of the times they are not there.’ (Interview 16, Special Education Teacher, Male)

### A heavy workload for the teachers

Teachers felt overworked. They noted that children with ASD needed much attention, yet most of the time they had large classes comprising of children with different functional abilities. Moreover, some of these children had very challenging behaviours making it difficult for the teachers to provide them instructions:

‘One of the challenges is that you need to have great care, so the teacher must pay a lot of attention to those children, so a lot of time is needed to take care of that child.’ (Interview 1, NGO Staff, Male)

‘There is a bit of a challenge for us since we teach in integrated classrooms, where some children are high functioning while others one (e.g. autistic child) is in his/her own world. So, we are forced to group learners according to their ability levels ….’ (Interview 3, Special Education Teacher, Female)

‘There are some behaviours that sometimes are extreme that are hard for me to cope with … So sometimes I fail to understand and overcome the extreme behaviour (aggressive behaviour, hyperactivity). That is a challenge that I have.’ (Interview 13, Special Education Teacher, Male)

### Lack of adequate support

It was reported that the teachers of children with ASD did not receive adequate support from the key stakeholders, for example, clinicians and NGO staff members. This lack of support comes in various forms such as focus on the ‘more visible’ disabling conditions, such as vision and hearing impairments:

‘What happens in special education, they look for those with deficits, most of which I think are either visual or hearing and speech.’ (FGD, Clinical Officer, Participant 1, Male)

Additionally, both teachers within the same schools and parents were perceived by some of those we interviewed as posing a challenge as some did not provide support to the teachers of children with ASD:

‘Another challenge is cooperation from other stakeholders. You can see that some caregivers are not cooperative, other teachers whom they teach with, the perception of these cases, some shy off, neglecting them, they do not even support them, morally or even materially (with classroom materials like pen, paper). These are challenges which they meet in the field.’ (Interview 14, Educationalist, Male)

‘Another challenge I think a teacher might face is the support from the caregivers. The caregivers leave these children to the teachers (in boarding schools) and forget about them, the constant monitoring of the child is absen.’ (Interview 1, NGO Staff, Male)

## Discussion

This study set out to examine the challenges faced by children with ASD and their teachers and parents. Interviews and FGDs identified numerous challenges: an absence of systematic identification and diagnostic procedures, improper placement, lack of trained personnel, shortage of learning materials and a lack of social support for the caregivers.

This study indicates that there were no systematic or multidisciplinary approaches to identifying and diagnosing the children who are labelled and placed in special units for having ‘ASD’; this is a problem that is shared with many other low- and middle-income countries (Olusanya et al. 2018). The danger with the current educational diagnosis and placement is the potential for errors in diagnosis, where children who may not be on the ASD spectrum may find themselves placed in the schools for children with ASD, whilst those who are on the spectrum may lack the care they need. Based on the interviews and FGDs carried out, it seems likely that a significant number of children currently labelled as having ASD may be experiencing other neurodevelopmental disorders and/or other intellectual disabilities. The study results emphasise the urgent need for developing measures and guidelines for the identification and placement of children with ASD and other neurodevelopmental disorders in the Kenyan education system. Early and accurate detection of children with ASD is crucial as earlier intervention promotes better prognosis (Fuentes et al. 2021; Lipkin et al. 2020). The measures to be developed need to be culturally appropriate, as this has been observed to be an important aspect of ASD diagnosis (DeWeerdt 2012).

In the management of children with ASD and other neurodevelopmental disorders, early diagnosis, placement and proper remediation may potentially be key in ensuring enhanced long-term prognosis (Lipkin et al. 2020; Mozolic-Staunton et al. 2020). The study findings reveal that these factors remain highly neglected in Kenya’s education setting, which may contribute to the worsening of these children’s conditions and their inability to achieve their developmental and educational potentials. There is, therefore, a need for policymakers and educationalists to consider ways of addressing this inadequacy in services.

Many educational challenges are reported by the population we interviewed. The most common (based on the number of times raised) was the lack of training for both educationalists and teachers who are supposed to conduct the diagnosis, place and educate the children. The adverse impact of children having to be attended to by unskilled personnel cannot be overestimated. Studies indicate that effective teaching is a key component to successful childhood outcomes for any children, and especially so for children with disabilities (Simpson, Mundschenk & Heflin 2011). In a review of literature on the impact of effective teaching, it was observed that children who had an ineffective teacher for three consecutive years performed 50 percentile points lower than peers of comparable abilities and skills who were taught by an effective teacher (Simpson et al. 2011).

Many of the challenges we observed had also been reported in a published work from Nairobi, where one of the interviewees said:

‘Families with children living in rural areas are more likely to raise a child with autism without ever receiving a proper diagnosis, and even if they did, it is likely there would not be a treatment centre located near their home. If a school in a rural part of our country does happen to have a special unit, this classroom will most probably be full of children, each with a unique disability, taught by a teacher with minimal training in what we call “special needs education”. These teachers are unable to give each child the individualized lesson plans they will require to learn and succeed, despite the best intentions of the teaching staff.’ (Interview A8) (Riccio 2011:8)

The congruence between the results from different parts of the country implies that there is an urgent need to take steps targeted at addressing these challenges. The challenge on placement warrants further discussion. Who and how to educate children with ASD remains a topic of continuing debate, even in resource-rich settings with longer histories of service provisions (Simpson et al. 2011); this indicates the complexities of the issues involved. In many regions of the world, the trend is towards inclusive education (i.e. having children with ASD learning in the mainstream classes). However, as discussed widely in the literature, successful inclusive education requires contextual consideration on how this would be implemented, including the move beyond inclusion to equity and the place of special education in this shift (Florian 2019). It would also require investment in preparing teachers adequately, taking care to instruct within the remits of each child’s functional abilities and possibilities for extra individualised care in the form of individual education plans, where needed. Moreover, teachers must be provided with an adequate working environment, especially ensuring that the teacher–student ratio is optimal so that the teachers can adequately instruct the children. Therefore, in settings such as ours, the development of appropriate facilities for children with ASD and their families, needs to take into consideration individual needs, and be based on extended dialogue between the different stakeholders.

The current educational system places a heavy burden on teachers who may be overwhelmed with educating children; they are not well-prepared to educate. Having large classes of children with different educational needs makes it difficult for them to meet the needs of each child within the classroom context. This situation not only results in underperformance but might also lead to burnout and emotional stress amongst teachers. Teachers of children with special needs are already at an elevated risk of experiencing burnout and stress; this is exacerbated by factors such as poor self-efficacy (i.e. a lack of confidence in one’s ability to meet their responsibilities) (Wisniewski & Gargiulo 1997). Research evaluating the impact of the burden on teachers’ emotional well-being and job performance is warranted to understand the kinds of programmes that should be put into place to address the needs of the teachers and to enhance their well-being and productivity.

### Limitations

This study focused only on three administrative locations within the coastal regions of the country, which may potentially limit the generalisability of our findings. As the study results indicate, there are potentially many problems with the diagnostic procedures in the Kenyan Coastal system. This means that we possibly have many children with other neurodevelopmental disorders; other children showing autistic traits, especially those with mild and moderate symptoms, may not be in the educational setting.

## Conclusion

This study highlights some of the gaps in the educational sector, especially at the Kenyan Coast, regarding children with ASD. There is an urgent need for evidence-based identification, placement and rehabilitation services to be put in place to meet the educational needs of children with ASD and other neurodevelopmental disorders.
